# Epigenetic Alterations in Meningiomas—A Review

**DOI:** 10.3390/biomedicines14091984

**Published:** 2026-09-03

**Authors:** Reinhold Nafe, Eike Steidl, Elke Hattingen

**Affiliations:** Institute of Neuroradiology, University Hospital, Goethe University Frankfurt, 60596 Frankfurt/Main, Germany

**Keywords:** meningiomas, DNA methylation, methylome analysis, histone modification, chromatin remodeling complexes, non-coding RNAs, micro-RNAs, circular RNAs

## Abstract

While genetic alterations in meningiomas have long been the subject of research, the diagnostic and prognostic significance of epigenetic alterations in this most common type of human brain tumor has become increasingly evident in recent years. The most significant findings to date have been DNA methylation analyses and the resulting classification of meningiomas into statistical clusters with different prognoses and various prevailing molecular and phenotypic characteristics. Even the loss of trimethylation of lysine at position 27 in histone 3 (H3K27me3) is of major importance, as it is significantly associated with more aggressive tumor behavior and a poorer prognosis. Since there are far more than just these epigenetic aspects associated with meningiomas, and given the clinical significance of this tumor type, a comprehensive review is needed that reflects the current state of knowledge. We conducted a systematic literature search in PubMed using broad search terms covering all epigenetic modifications and all subcategories. The search was not limited to a specific time frame. The 513 references identified in this manner were fully analysed. To ensure a comprehensive review that covered all relevant topics and reflected the current state of knowledge, 196 references were included in the reference section. In addition to DNA methylation and forms of histone modification, the review includes known alterations in chromatin remodeling complexes, as well as alterations in various types of non-coding RNAs, such as microRNAs and circular RNAs. Moreover, the discussion also addresses epigenetic modifications relevant to oncology that have not yet been identified or investigated in meningiomas. Therefore, this review additionally provides an appropriate overview of epigenetic mechanisms and alterations in general. The broad spectrum of epigenetic alterations opens up far-reaching prospects for future forms of molecular therapy in patients with meningiomas; the current state of epigenetic molecular therapeutic approaches for meningiomas will be addressed in the final section of the discussion.

## 1. Introduction

A comprehensive review of epigenetic alterations relevant to oncology must cover four topics, namely, (1) DNA methylation; (2) histone modifications such as histone methylation, acetylation, and phosphorylation; (3) alterations in chromatin remodeling complexes; and (4) changes in the expression of non-coding RNAs, with microRNAs, long non-coding RNAs, and circular RNAs being the most well-studied. Such a review is particularly important for two reasons, especially given that meningiomas are the most common type of brain tumor in humans. First, epigenetic characteristics and alterations contribute significantly to a clinically valid classification of meningiomas with prognostic relevance that goes beyond a prognostic assessment of individual cases based exclusively on tumor grading [1,2,3]. Furthermore, the spectrum of epigenetic alterations, including aspects that have not yet been investigated in meningiomas, opens up far-reaching prospects for the development of new molecular therapeutic approaches. For this reason, the current findings on meningiomas regarding the four epigenetic aspects mentioned above are presented in detail, including those aspects whose clinical and prognostic significance is still only partially understood and which require further investigation. In contrast to the many molecular therapeutic approaches that have been investigated to date in vitro, experimentally, and in clinical trials for meningiomas [4,5,6], epigenetic approaches for this tumor type are largely limited to the use of histone deacetylase inhibitors [1,7,8]. However, there are also reports of other therapeutic approaches in this area [9]. This overview is intended to serve as a comprehensive framework for the further development of diagnostic and therapeutic strategies, which should be based on the individual molecular and, consequently, epigenetic characterization of each tumor case.

## 2. DNA Methylation

### 2.1. General Aspects

This is the most extensively studied epigenetic modification in diseases. Although covalent binding of a methyl group to various sites on a nucleic acid base and to different bases have been described, the term “methylation” is often used in a narrower sense to refer to the binding of a methyl group (CH3) to the 5’-position of a cytosine base [10]. This primarily concerns the cytosines of CpG sites (cytosine–phosphate–guanine sites), with approximately 70% of all CpG sites in mammalian DNA being methylated under physiological conditions [11]. Physiologically, DNA methylation is involved in the regulation of embryonic development, X-chromosome inactivation, and genomic imprinting. In pathology, the methylation status of gene promoter regions is of particular importance, as their hypermethylation leads to the silencing of the affected gene, while demethylation results in its activation. The opposite is true in the gene body, where the degree of DNA methylation positively correlates with gene expression. Through experimental demethylation of the gene bodies, downregulation of the respective genes could be confirmed [12]. The dominant role of promoter regions in tumor research is directly demonstrated by the evidence of a correlation between the degree of (hyper)methylation and the silencing of the gene in question, which can be confirmed by additional expression and mRNA analyses. Overall, various studies on DNA methylation agree that hypermethylation of a gene and its reduced expression are highly correlated [10,11,13,14]. To date, DNA methylation has primarily been detected using microarrays following bisulfite treatment. As a result of the specific conversion of unmethylated cytosine to uracil, the remaining cytosine corresponds to methylated cytosine and can be detected directly using the arrays. In routine diagnostic practice, the prerequisite for a reproducible analysis is the availability of at least 100 ng of bisulfite-treated DNA and a minimized content of non-tumor tissue in the sample being tested [10]. Several new technical developments are worth mentioning, in particular, new microarrays capable of analysing over 900,000 CpG sites; this method is currently being further developed and tested [15,16]. Second, the increasing use of nanopore sequencing for methylation analysis should be noted [17]. Third, so-called enzymatic methyl sequencing must be mentioned, in which bisulfite pretreatment is replaced by treatment with the enzymes methylcytosine dioxygenase and apolipoprotein B mRNA editing catalytic polypeptide-like 3A (APOBEC3A), which helps to better preserve DNA integrity during sequencing [18]. These innovations underscore the growing importance of methylation analysis in oncology in the future. With regard to the methylome analysis in meningiomas, two fundamental methodological goals have been pursued to date: First, the development of methylome-based unsupervised classification methods that correlate with clinical outcome and patient prognosis. These methods are each based on the analysis of a large number of genes. Second, many research groups have also emphasized the importance of the methylation status and, consequently, the activation status of individual genes.

### 2.2. Classification of Meningiomas Based on Methylome Analysis

The significance of multivariate statistical classification of meningiomas using data from a methylome analysis lies, firstly, in the prognostic differences between the individual clusters generated, and, secondly, in the identification of further differences among these clusters with regard to the frequencies of specific genetic alterations, the prevalence of certain tumor locations, and the histological subtypes of meningiomas. From a large series of 497 meningiomas, six statistically distinct clusters were generated, the first three of which included cases with a good prognosis and a long progression-free survival (PFS) (MC ben-1/2/3). The fourth and fifth groups (MC int-A/B) included cases with statistically shorter PFS, while the sixth group (MC mal) comprised cases with a poor prognosis and only short PFS [3]. The tumors in the MC ben-2 group are characterized by the absence of aberrations on chromosome 22, by the predominance of secretory and meningothelial-type meningiomas, and by a predominantly basal location at the base of the skull. All other groups showed chromosome 22 aberrations and/or a mutation in the NF gene and a predominant location at the convexity [3]. Subsequently, several research groups conducted further cluster and regression analyses, and the authors of these studies have highlighted the far-reaching common findings [1,2,14,19,20,21]. One key finding is that NF2 aberrations can occur not only in groups with intermediate and poor prognoses but also in tumors from a group with a good prognosis. Another important finding is the limited prognostic significance of the tumor grade as an isolated prognostic criterion: many WHO Grade 1 tumors were classified into higher clusters in line with their poorer clinical outcomes, and conversely, some meningiomas with higher tumor grade were classified into lower clusters [14]. In particular, for atypical meningiomas with tumor grade 2, methylation analysis combined with other genetic alterations is considered an additional benefit, since methylation analysis reveals that atypical meningiomas can be found in both the clusters with a good prognosis and those with a poorer prognosis [3,22]. One study group developed a specific approach to methylome analysis based on classification into four clusters, whose names directly reflect the upregulation of dominant signaling pathways. The tumors in the second group are characterized by the NF2 wild-type; the tumors in the first group are characterized by upregulation of immunogenic signaling pathways, a good prognosis, and an enrichment of macrophages in the tumor tissue. The third group shows upregulation of metabolic signaling pathways converging on nucleotide and lipid metabolism, while the fourth group, which has the poorest prognosis, is characterized by the dominance of activated cellular proliferation signaling pathways [1,2]. One key finding of this study group is the prognostic significance of methylation analysis itself. This was confirmed by a statistical approach using unsupervised classification applied to 1698 meningiomas, in which only data from the methylation analysis were used. The validation of this cohort yielded results identical to the previously developed classification of meningiomas into the four clusters “immunogenic”, “NF2-wildtype”, “hypermetabolic”, and “proliferative” [2]. The findings of this research group, as well as those of other studies, demonstrate that, in addition to the unsupervised classification of tumors, research into the significance of the methylation status of individual genes in meningiomas is also important. This research approach has been pursued even before the classification methods presented above were developed.

### 2.3. Research on the Methylation Status of Individual Genes

Some of the genes individually examined in meningiomas showed a hypermethylated status (Table 1a), but also, hypomethylation is notable in a number of other genes that were investigated (Table 1b). An important tumor suppressor is WNK lysine deficient protein kinase 2 (WNK2), an inhibitor of the epidermal growth factor receptor (EGFR). WNK2 is hypermethylated in most of the atypical (81%) and anaplastic (71%) meningiomas examined with tumor grades 2 and 3 [23]. Reactivation of WNK2 using a methylation inhibitor in vitro prevented colony formation, confirming WNK2’s function as a tumor suppressor. Furthermore, a comparison with other tumor types showed that WNK2 was hypermethylated in the vast majority of meningiomas but only in isolated cases of other tumor types, suggesting that WNK2 methylation is largely tumor-type-specific to meningiomas [23]. Several studies have examined additional tumor suppressor genes whose hypermethylation is common in meningiomas with tumor grades 2 and 3, but less common in cases with tumor grade 1. This applies to the genes of the tissue inhibitor of metalloproteinase 3 (TIMP3), glutathione S-transferase Pi 1 (GSTP1), tumor protein P73 (TP73), maternally expressed 3 (MEG3), Proenkephalin (PENK), and mal-T cell differentiation protein 2 (MAL2) [24,25,26,27]. The analysis of homeobox genes (HOX genes) revealed a notable finding in meningiomas, namely hypermethylation of four genes from the homeobox A cluster *(HOXA5*, *HOXA6*, *HOXA9*, and *HOXA11*), while the other HOX genes from clusters B, C, and D showed no hypermethylation (Table 1a). According to the authors, this finding suggests a correlation between concordant methylation in the HOXA cluster and tumorigenesis in meningiomas [28]. Hypermethylation of the *HOXA6* and *HOXA9* genes in meningiomas was also confirmed in a later study [24]. An important contribution regarding the hypomethylation of genes is a study that compares the methylation status of meningiomas and the dura mater in paired samples. In contrast to the dura, meningiomas were found to exhibit hypomethylation of the craniofacial patterning transcription factor forkhead box C1 (FOXC1) and its upstream target FOXC1 upstream transcript (FOXCUT) (Table 1b). This hypomethylation was detectable in the tumors in all pairwise comparisons, as was increased expression of the mRNA and the protein. The authors emphasize the need for further research into the role of FOXC1 and FOXCUT in meningiomas; it is known that they activate the PI3K/AKT signaling pathway in tumors [29]. Hypomethylation has also been detected in another gene of the Forkhead Box family, FOXM1; however, this is specifically observed in the methylation class of proliferative meningiomas with a poor prognosis [1]. Two genes from the human leucocyte antigen (HLA) family, *HLA-DMA* and *HLA-DPB1*, also showed hypomethylation in a specific methylation class, namely that of immunogenic meningiomas. In addition, hypomethylation was found in immunogenic meningiomas for three genes involved in the activation of immunogenic cells and interaction with extracellular matrix components: The genes from lymphatic vessel endothelial hyaluronan receptor 1 (LYVE1), C-C motif chemokine ligand 21 (CCL21), and CD3 epsilon subunit of the T-cell receptor complex (CD3E) (Table 1b). The authors call for further investigation of the methylation classes described above with regard to specific genomic and epigenetic characteristics, including the identification of therapeutic vulnerabilities as a basis for new treatment approaches for meningioma patients [30].

**Table 1:** (**a**) Individual genes with **hypermethylation** in meningiomas; abbreviations, gene locus, and full names of the encoded proteins; physiological gene functions (blocked due to promoter hypermethylation) [23,24,25,26,27,28]. (**b**) Individual genes with **hypomethylation** in meningiomas; abbreviations, gene locus, and full names of the encoded proteins; physiological gene functions (enhanced due to promoter hypomethylation) [1,29,30].

(**a**)		
**Gene, Locus**	**Full Name of the Encoded Protein**	**Physiological Functions (Blocked Due to Promoter Hypermethylation of the Gene in Meningiomas)**
***WNK2*** (9q22.31)	WNK lysine deficient protein kinase 2	Regulation of electrolyte homeostasis, cell signaling, survival, and proliferation
***TIMP3*** (22q12.3)	Tissue inhibitor of metalloproteinase 3	Tissue remodeling, anti-angiogenic effect, suppression of tumor progression
***GSTP1*** (11q13.2)	Glutathione S-transferase Pi 1	Detoxification of lipid peroxidation products, protection against oxidative stress
***TP73*** (1p36.32)	Tumor protein P73	Induction of cell cycle arrest, DNA repair, and apoptosis
***MEG3*** (14q32.2)	Maternally expressed 3	Suppression of cell growth and inhibition of tumor cell proliferation
***PENK*** (8q12.1)	Proenkephalin	Modulation of pathways relevant to analgesic and sensory regulation, promotion of apoptosis and inhibition of tumor growth
***MAL2*** (8q24.12)	Mal-T cell differentiation protein 2	Involved in moving membrane-bound proteins through cells, known properties as a tumor promoter or suppressor in different tumor types
***HOXA5/6/9/11*** (7p15.2)	Homeobox A 5/6/9/11	Involved in the regulation of cellular and tissue-specific differentiation along the body axis, together with all other HOX genes
(**b**)		
**Gene, Locus**	**Full Name of the Encoded Protein**	**Physiological Functions (Enhanced Due to Promoter Hypomethylation of the Gene in Meningiomas)**
***FOXC1*** (6p25.3)	Forkhead box C1	Involved in the formation of mesoderm-derived tissues, the gene is active in developmental and disease-related pathways
***FOXM1*** (12p13.33)	Forkhead box M1	Promotes cell proliferation by regulating genes that support progression through the cell cycle, supports DNA repair
***HLA-DMA*** (6p21.32)	Major histocompatibility complex, class II, DM alpha	Promotes peptide loading onto class II molecules, its expression correlates with increased immune cell infiltration in tumors
***HLA-DPB1*** (6p21.32)	Major histocompatibility complex, class II, DP beta 1	Involved in the presentation of exogenous peptides on antigen-presenting cells and activation of CD4+ T cells
***LYVE1*** (11p15.4)	Lymphatic vessel endothelial hyaluronan receptor 1	Binding of hyaluronan, functions as a receptor at the cell surface, involved in interactions with extracellular matrix components and transport-related processes
***CCL21*** (9p13.3)	C-C motif chemokine ligand 21	Recruitment of naive T cells and dendritic cells to secondary lymphoid organs, stimulation of chemotaxis of activated T cells
***CD3E*** (11q23.3)	CD3 epsilon subunit of T-cell receptor complex	Essential for T-cell development and adaptive immunity, involved in the activation of ERK, NF-κB, and calcium signaling pathways

## 3. Histone Modifications

### 3.1. General Aspects

Histones are highly conserved proteins involved in the condensation of cellular DNA. In this process, a DNA segment with a total length of 147 base pairs is wound exactly 1.65 times around the nucleosome, which consists of an octamer of a total of eight core histones (H), namely two copies each of H2A, H2B, H3, and H4 [31]. In addition, the nucleosome contains the two linker histones H1 and H5, which ensure the connection and integrity between adjacent nucleosomes and the DNA wound around them [31,32]. This packaging of DNA enables the regulation of gene expression through epigenetic activation or silencing without altering the DNA sequence. There are many different types of post-transcriptional modifications of histones, particularly involving H3 and H4, and new ones are continuously being described [33,34]. Under physiological conditions, these modifications influence the regulation of the activity of the relevant gene regions. Pathological modifications, on the other hand, can influence the pathophysiology of diseases or tumors, potentially leading to reduced control of the cell cycle, increased invasiveness, or a higher incidence of tumor recurrence [34,35]. Alterations in histone methylation have been detected in meningiomas. Furthermore, there is also evidence suggesting a role for histone acetylation in meningiomas due to altered expression patterns of histone deacetylases (HDACs) and histone acetyltransferases (HATs), as well as a role for the histone phosphorylation PHH3.

### 3.2. Histone Methylation

The most clinically significant histone methylation in meningiomas is the trimethylation of lysine at position 27 on histone H3 (H3K27me3). The immunohistologically detectable absence of this methylation in meningioma tissue is significantly associated with a poorer prognosis and a higher incidence of recurrence compared to patients with retained H3K27me3 in the tumor tissue [35,36,37]. A separate analysis of tumor grades 1 through 3 shows that the proportion of meningioma cases with loss of H3K27me3 increases as the tumor grade rises. Specifically for grade 2 meningiomas, several studies have identified a significant difference in prognosis between patients with and without loss of H3K27me3 as measured by the frequency of recurrence or the duration of progression-free survival [37,38,39]. Even in Grade I tumors, a marginal prognostic difference appears to emerge; in contrast, however, several studies found no prognostic difference specifically in Grade III anaplastic meningiomas when cases with and without loss of H3K27me3 were compared [37,38,39,40]. A study encompassing a considerable number of 47 anaplastic meningiomas came to a different conclusion: 10 cases (21%) showed a loss of H3K27me3 and a significantly shorter survival time. However, more than half of all cases examined had a history of a lower grade meningioma at the same site, so that most of the anaplastic meningiomas examined were considered recurrent tumors [41]. Therefore, particularly in the case of anaplastic meningiomas, further studies are needed to determine whether the loss of H3K27me3 has prognostic significance specifically for this group.

To date, the mechanisms underlying this specific histone methylation and its pathological loss remain the subject of research. It is hypothesized that meningioma recurrence itself acts as a triggering factor for the loss of H3K27me3, and that, more generally, metabolic shifts associated with tumor progression such as enhanced glycolytic flux, lactate accumulation and activation of lysine demethylases promote the depletion of H3K27me3 [33,42]. It is also suspected that radiation therapy may influence the decline in H3K27me3, thereby directly inducing an increased risk of recurrence [31,35]. What is certain is the direct role of the polycomb repressive complex 2 (PRC2) together with its catalytic component, the histone methyltransferase enhancer of zeste Homolog 2 (EZH2), in the formation of the triple methylation H3K27me3. In contrast, the methyl groups are removed by the two demethylases KDM6A and KDM6B [35,42]. The exact nature of the dysregulation of all these components in meningiomas is not yet fully understood; studies on other types of tumors have yielded conflicting results [31,35]. In particular, the role of H3K27me3 varies depending on the type of tumor. For example, the immunohistochemical detection of H3K27me3 and EZH2 correlates with a poorer prognosis of IDH-mutant astrocytomas [43]. In contrast, in pediatric posterior fossa ependymomas type A, the loss of H3K27me3 is associated with a poorer prognosis compared with posterior fossa ependymomas without loss of H3K27me3 (Type B) [44]. These signaling pathways involving EZH2 and the loss of H3K27me3 have not yet been analysed in detail in meningiomas [37]. Among other alterations in histone methylation in tumor biology, the trimethylation of lysine at position 4 of histone 3 (H3K4me3) is particularly noteworthy. Unlike H3K27me3, H3K4me3 is associated with an increase in the transcriptional activity of the affected gene regions [33,45]. In pediatric high-grade gliomas, this form of histone methylation is associated with increased tumor proliferative activity [46]. Recent research was conducted on the histone methyl transferase euchromatic histone methyl transferase 2 (EHMT2, syn. G9a) and other methyltransferases that belong to the Jumonji domains, which are overexpressed in many tumors. Specific inhibition of these methyl transferases led to significantly reduced cell viability in meningioma cells in vitro [9]. EHMT2 catalyses the di- or trimethylation of lysine at position 9 of histone 3 (H3K9me2/3), which leads to the inhibition of tumor suppressor genes and thus promotes tumor growth. In contrast, experimental inhibition of EHMT2 leads to the activation of apoptosis, a reduction in heat-shock-protein expression, and a decrease in the tumor growth of meningiomas [47]. Therefore, it is hypothesized that EHMT2/G9a and, consequently, the histone modification H3K9me2/3 could be another promising therapeutic target for meningiomas [9,47].

### 3.3. Evidence Suggesting the Significance of Histone Acetylation in Meningiomas

Pathological changes in histone acetylation can result from dysregulation of histone deacetylases (HDACs) or histone acetyltransferases (HATs). Their significance in tumor biology has emerged from the realization that application of histone deacetylase inhibitors (HDACis) leads to inhibition of proliferation and tumor growth in many tumor types, with an improvement in clinical prognosis. HDACs are classified into several classes based on their homology to different yeast strains and their cofactors (zinc or NAD+), and most HDAC inhibitors (HDACis) equally inhibit numerous HDACs from different classes [48]. Therefore, it is presumed that the effect of HDACis is largely based on the reduction in the blocking effect of several HDACs on tumor suppressor genes [49]. While HDAC inhibitors are already being tested and used in clinical trials for certain types of tumors, such as T-cell lymphomas and gliomas [48], research on meningiomas is still in the preclinical stage. The histone deacetylase AR-42 has been tested in individual cases of NF2-associated schwannomas and meningiomas, with evidence of inhibited tumor growth and downregulation of the AKT/PIK3 pathway in most tumors. However, the authors emphasize the need for further experimental and clinical studies [50,51]. Noteworthy is the experimental combination of oncolytic herpes simplex virus therapy (oHSV) with the pan-HDAC inhibitor panobinostat in a mouse model with xenografts of human meningiomas. This combination resulted in enhanced tumor growth inhibition compared to oHSV therapy without panobinostat [52]. Administration of the HDAC inhibitor vorinostat resulted in decreased viability of human meningioma cells in vitro, as well as reduced tumor growth and prolonged survival in an experimental model using intracranial xenografts of human meningiomas. However, this therapeutic effect of vorinostat was observed specifically in the case of meningiomas with strong activation of proliferative signaling pathways (M4), but not in other meningioma subtypes defined by the authors, such as tumors with pronounced activation of immunogenic or metabolic signaling pathways. The authors therefore suspect differences in the efficacy of HDACis across different molecular subgroups of meningiomas, a finding that requires confirmation in further studies [37]. HDAC6 is a histone deacetylase that is overexpressed in many types of tumors, including human meningiomas. In an in vitro study of meningioma cells, an even greater increase in HDAC6 expression was observed following irradiation of the tumor cells, which in turn was prevented by administration of the HDAC inhibitor Cay10603 prior to irradiation. The combination of Cay10603 and subsequent irradiation resulted in a particularly pronounced toxic effect on the meningioma cells. The authors confirmed that the cause was the increased degradation of β-catenin and the resulting inhibition of the tumor-promoting WNT signaling pathway [53]. Sirtuins are histone deacetylases that, unlike other HDACis, require NAD+ rather than zinc as a cofactor. They are often referred to as “aging-associated genes” and regulate a variety of signaling pathways involved in cell proliferation, the immune system, and tumor suppressor genes such as p53 [54]. Evidence of a role for sirtuins in meningiomas was found in the study cited above regarding EHMT2, whose experimental inhibition led to reduced expression of heat shock proteins due to decreased expression of SIRT1 [47]. Further evidence is the dysregulation of SIRT1 expression in several immunogenic cells in meningiomas compared to non-neoplastic tissue. The authors conclude that immunogenic dysregulation involving the sirtuin SIRT1 contributes to the development of human meningiomas; they emphasize the need for further experimental research into the causal relationships and the specific role of SIRT1 [54].

Histone acetyltransferases (HATs) are classified based on their sequence homologies, similar to deacetylases; a second classification distinguishes between HATs in the cell nucleus (Type A) and HATs in the cytoplasm (Type B), which acetylate newly synthesized histones before they are transported into the cell nucleus. Additionally, it is important to note that HATs also acetylate lysine of many non-histone proteins and are therefore often generally referred to as “lysine acetylases” [55]. In contrast to the growing importance of HDACs and their therapeutic inhibition by HDACis in tumor biology, comparatively little is known about the role of individual HATs. The histone acetylase that has been most extensively studied in the field of oncology is HAT1, a type B acetylase that acetylates newly synthesized H4 histones at lysine 5 and lysine 12. In a HAT1 knockout mouse model, the importance of HAT1 in maintaining genomic stability was confirmed, as well as its role in the acetylation of additional lysine positions on histone H3 [56]. Immunohistochemical and molecular analysis of human meningiomas, oligodendrogliomas, and gliomas revealed, in the case of meningiomas and gliomas, increased expression of HAT1 in tumors with higher tumor grades. Due to the short follow-up period for the patients, no correlation with survival time was assessed. According to the authors, these results provide new evidence for HAT1 as a potential marker and a possible therapeutic target for primary brain cancers [57]. Taken together, the altered expression of various HDACs and HAT1 in meningiomas provides strong evidence of a corresponding alteration in the influence of these HDACs and HAT1 on histone acetylation, such as the modification of histone 4 acetylation resulting from HAT1 overexpression [56].

### 3.4. Evidence for the Prognostic Significance of Histone Phosphorylation PHH3 in Meningiomas

**Figure 1:**
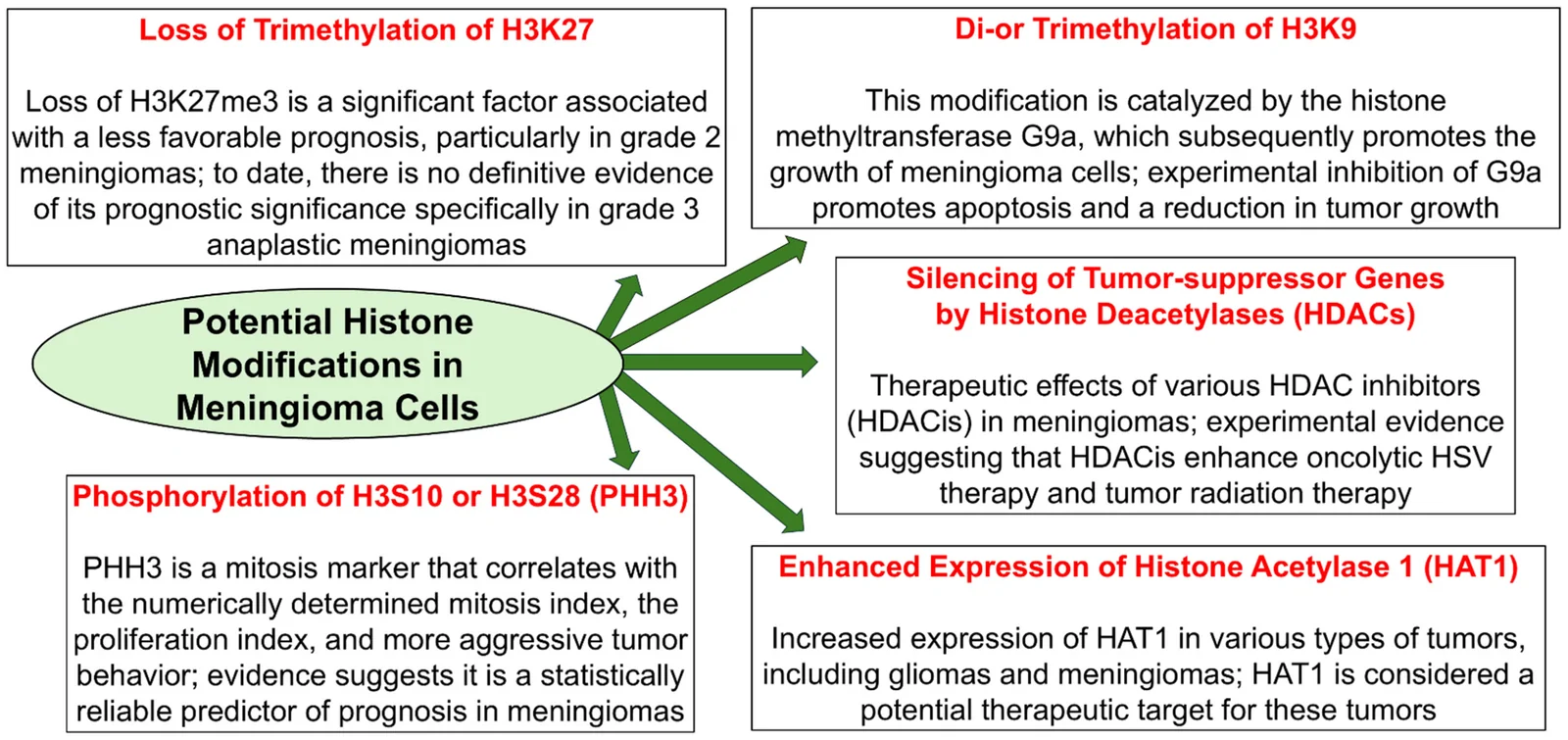
Potential epigenetic histone modifications in meningioma cells as described in the text. In addition to loss of H3K27me3, current findings also indicate that the di- or trimethylation of lysine 9 on histone H3 (H3K9me2/3) is a prognostically unfavourable modification in meningiomas, as is the silencing of tumor suppressor genes by histone deacetylases (HDACs), the increased expression of the histone acetylase HAT1, and a high mitotic index, as indicated by the intensity of serine phosphorylation at positions 10 or 28 of histone H3 (PHH3) [7,35,36,37,38,39,40,41,42,48,49,50,51,52,53,54,56,57,58,59,60].

Regarding histone phosphorylation, one form is particularly significant in tumor biology in general: the phosphorylation of histone H3 at serine 10 or serine 28. This phosphorylation (PHH3) is specific to mitosis and does not occur during interphase [58]. Using various immunohistochemical antibodies specific for phosphorylated serine 10 or serine 28, a high correlation of the number of PHH3-positive meningioma cells was demonstrated with the numerically determined mitotic index in hematoxylin/eosin sections, with the Ki67 proliferation index, as well as with a poorer prognosis and shorter time to recurrence [59,60]. No significant differences were detected between the various PHH3 antibodies [60]. Notably, in the multivariate Cox analysis, only the PHH3 index was an independent predictor of time to recurrence, whereas this was not the case for the HE mitotic index or the Ki67 proliferation index (Figure 1) [60]. Little is currently known about the molecular mechanism of PHH3. As expected, the numerical occurrence of PHH3-marked tumor cells correlates with prognosis and the degree of tumor proliferation; however, a study that took into account the expression of the transcription factor STAT3 showed no correlation with PHH3 in meningiomas [61]. In addition to histone methylation, acetylation, and phosphorylation, other forms of histone modification include SUMOylation, citrullination, lactylation, and crotonylation [33,34,62]. Although these other forms of histone modification have not yet been described in meningiomas, it is nevertheless useful to discuss them in this review, as these four forms play a role in several tumor types. Therefore, they are addressed in the discussion section. The potential significance of these forms of histone modification in meningiomas should also be explored in the future.

## 4. Alterations in the SWI/SNF Chromatin Remodeling Complexes

### 4.1. General Aspects

The mammalian SWI/SNF complexes are ATP-dependent chromatin remodeling complexes that control genomic architecture and histone conformation to regulate gene expression, proliferation, and cellular differentiation. Due to their influence on DNA and histones, these complexes therefore constitute an epigenetic regulatory mechanism of the mammalian cell alongside DNA methylation, histone modification, and the action of non-coding RNA molecules. The complicated naming convention stems from the discovery of the *yeast* genes “Switched” (SWI) and “Sucrose non-fermentable 1/2” (SNF1/2), which play a role in yeast in the regulation of gene expression and glucose utilization [63,64]. Shortly thereafter, the homologous “Brahma gene” (BRM) was discovered in the *Drosophila fruit fly*, which exhibits a similar gene-regulatory function [65]. The two homologous genes in mammals are the “SWI/SNF-related, matrix-associated, actin-dependent regulator of chromatin A2” (SMARCA2), also named “BRM” after the Brahma gene, as well as the “Brahma-related gene 1” (BRG1, SMARCA4) [66]. Due to the high degree of similarity between these genes across different species, the synonymous term “Brahma-associated factor complex” (BAF complex) is also frequently used for the SWI/SNF complexes in mammals [67].

The three mammalian SWI/SNF complexes—the canonical BAF complex (cBAF), the polybromo-BAF complex (PBAF), and the non-canonical BAF complex (ncBAF)—are composed of a total of 29 different proteins. Of these proteins, SMARCA2/4, SMARCC1, SMARCCD1, ACTB, ACTL6A/B, and BCL7A-C are present in all three complexes [68]. The two ATPases SMARCA2 (BRM) and SMARCA4 (BRG1) are mutually exclusive and can occur alternately in all three complexes. In line with the partially overlapping protein components, the functions of the three complexes also overlap and have so far been studied only to a limited extent [67,68,69]. cBAF and PBAF essentially function as tumor suppressors and are involved in DNA repair and the control of cell proliferation. In the case of the PBAF complex, it is known to be concentrated in the promoter regions of genes [70,71]. Unlike cBAF, the PBAF complex contains the protein that gives it its name, Polybromo1 (PBRM1) [72]. The functions of the non-canonical ncBAF complex are still largely unknown. Co-localization of ncBAF with the zinc-dependent CCCTC-binding factor (CTCF) is known; CTCF is distributed throughout the genome and plays a key role in maintaining the three-dimensional DNA structure [70]. Unlike cBAF and PBAF, ncBAF is thought to play only a minor role in tumor suppression; instead, it promotes cell growth and may exhibit oncogenic activity in certain types of tumors [70,71,73].

Similar to the overlapping functions of the cBAF and PBAF complexes in particular, the individual proteins of the BAF complexes also exhibit similar functions. It should be emphasized that the functionality of these proteins can only be fully understood within the overall context of the BAF complexes; clues to their respective functions are revealed in particular through gene mutations. According to a pan-cancer data analysis of over 13,000 cancer patients, the most frequently mutated gene regarding the components of the BAF complexes is that of the ARID1A protein (AT-rich interaction domain 1A) in 9% of all cases. The genes SMARCA4, ARID1B, ARID2, and PBRM1 also show frequent mutations, each in 3–4% of the cases examined, followed by the important tumor suppressor SMARCB1, which exhibits a mutation in about 1% of all cases [74]. Regarding mutations in components of BAF complexes in meningiomas, mutations in ARID1A, PBRM1, SMARCA4, and SMARCB1 are particularly significant. This also applies to mutations in SMARCE1, which occur in clear cell meningiomas but are rare in other types of tumors.

### 4.2. Mutations in Individual Components of the BAF Complexes

As in the pan-cancer study mentioned above [74], the cBAF-specific protein ARID1A (1p36.11) is frequently mutated in meningiomas of all tumor grades. In a cohort of 255 meningiomas, an ARID1A mutation was found in 19.1% of Grade 1 tumors, 16.8% of Grade 2 tumors, and 15.8% of Grade 3 tumors. According to the authors, this mutation represents a statistically independent predictor of more aggressive tumor behavior and shorter survival of patients with meningiomas [75]. Mutations of ARID1A can also lead to ovarian and endometrial carcinoma, bladder carcinoma, or gastric carcinoma [72]. Physiologically, ARID1A is involved in a variety of signaling pathways, such as telomerase activation, the WNT signaling pathway, and the PIK3 signaling pathway. In the context of tumorigenesis, particular importance is attached to the defective regulation of the PIK3 signaling pathway resulting from an ARID1A mutation [72,75]. The PBAF-specific protein polybromo 1 (PBRM1, 3p23.1) is less frequently mutated in meningiomas. In contrast, the highest frequency for PBRM1 mutations has been reported for renal cell carcinoma, this mutation occurs in about 30% of these tumors [74]. The first meningiomas described with a PBRM1-mutation were of the rhabdoid type and the papillary type [76,77]. Subsequently, additional meningiomas with this mutation were described, each of which was associated with an unfavourable clinical course and a poorer or delayed response to adjuvant therapy such as immunotherapy [78]. Furthermore, the co-occurrence of BAP1 mutations and PBRM1 mutations was observed in many cases [76,77,78]. One notable feature is that PBRM1 is considered a tumor suppressor, although it can also exert an oncogenic effect, with elevated mRNA levels observed in various types of tumors, such as prostate cancer [79,80]. Regarding the physiological function of PBRM1, it is known that its bromodomains are primarily involved in binding to histones with acetylation of lysine at position 14 (H3K14ac) [79,81]. The presence of a PBRM1 mutation is associated with impaired chromatin binding and dysregulation of signaling pathways associated with cell growth. Based on current knowledge, activation of the mTOR1 pathway as a result of a PBRM1 mutation can be considered certain [81]. Even in the current literature, the limited number of publications on the role of PBRM1 and its mutations in meningiomas is noted [78].

*SMARCB1* (22q11.23) is another one of the six BAF-related genes most frequently mutated in malignant tumors [74]. In meningiomas as well, a somatic mutation in the *SMARCB1* gene is one of the most common mutations among BAF-related genes. Meningiomas associated with this mutation tend to occur intracerebrally near the convexity, although spinal meningiomas have also been reported [82,83,84]. A systematic analysis of the frequency of this mutation across different tumor grades is still pending; it is hypothesized that the presence of *SMARCB1* mutations is associated with more aggressive behavior of the meningiomas [84,85]. A notable feature in meningiomas is the frequent co-occurrence of a *SMARCB1* mutation with an NF2 mutation due to the proximity of the two gene loci (22q11.23 and 22q12.2) and the associated involvement of both genes in a loss of this chromosomal region [82,83,84]. Another link to meningiomas is the possibility of a *SMARCB1* germline mutation, which can lead to a familial clustering of schwannomas and/or meningiomas. Although most schwannomas are sporadic tumors, some cases may have a familial origin, in which the cause involves not only a *SMARCB1* germline mutation but also inactivation of the adjacent LZTR1 gene (leucine zipper-like post-translational regulator 1) and the NF2 gene. In addition to cases involving schwannomatosis alone, some cases also present with a combination of schwannomas and meningiomas [83,86]. Functionally, SMARCB1 is a tumor suppressor protein that regulates the cell cycle by inducing CDKN2A expression and phosphorylation of the RB1 protein. In contrast, a SMARCB1 mutation leads to the activation of the PRC2 complex and, as a result, to increased trimethylation of H3K27me3 with silencing of chromatin at regulatory elements [87]. Nevertheless, it needs to be emphasized that the molecular impact of a *SMARCB1* mutation, particularly in meningiomas, is not fully understood. An important observation in SMARCB1-mutated meningiomas is the development of an immunosuppressive microenvironment due to the increased presence of anti-inflammatory CD163-positive macrophages [88].

A germline mutation in the *Brahma-related gene 1* (*SMARCA4*, syn. *BRG1*, 19p13.2) is the cause of rhabdoid tumor predisposition syndrome (RTPS) type 2. As in the case of RTPS type 1 resulting from a germline mutation in SMARCB1, malignant rhabdoid tumors of various locations also occur in type 2; however, there is no known association with meningiomas [83,89]. In contrast, somatic *SMARCA4* gene mutations are among the most common mutations associated with the BAF complexes in meningiomas. Whether the majority of meningiomas with this mutation tend to have an intraventricular location is not clearly evident from the literature; in some studies, no *SMARCA4* mutations were found in any of the tumors with intraventricular location [74,75,90]. Of the meningiomas described to date, most tumors with this mutation are grade 2, while fewer are grade 1. In some studies, no anaplastic meningiomas of WHO grade 3 were found with this mutation, whereas isolated anaplastic meningiomas were identified in other studies [75,85]. Functional cell studies have shown that a *SMARCA4* mutation leads to the suppression of glycolysis-associated genes and to increased oxygen utilization as a result of the upregulation of mitochondrial oxidative phosphorylation. Additionally, in non-small-cell lung cancer, inhibition of tumor cell death due to the inhibition of ferroptosis resulting from *SMARCA4* deficiency in the presence of a *SMARCA4* mutation has been demonstrated [81,91,92]. Whether the presence of a somatic mutation of *SMARCA4* has prognostic significance in meningiomas cannot yet be definitively confirmed.

Mutations in the *SMARCE1* gene (also known as BAF57, 17q21.2) are responsible for Coffin–Siris syndrome and clear cell meningioma (CCM). Coffin–Siris syndrome is characterized by generalized developmental delay and hypoplasia of the nail of the fifth finger. While in this syndrome a germline mutation of *SMARCE1* represents only one possible genetic alteration among other mutations of BAF-associated genes, in clear cell meningiomas, the *SMARCE1* mutation is the central genetic alteration [93]. A *SMARCE1* germline mutation in meningiomas was first detected in patients with spinal CCM; this mutation and the same tumor diagnosis were also identified in family members of these patients. None of the patients examined had an NF2 germline mutation [94]. Soon thereafter, *SMARCE1*-mutated CCM with intracranial localization were also described [95]. It is now known that CCM can occur both in association with a germline SMARCE1 mutation and with a sporadic SMARCE1 mutation. Penetrance in the case of a *SMARCE1* germline mutation is very low, so screening of patients every three years is recommended, even for asymptomatic patients with no evidence of a tumor [93,96]. CCMs are characterized by sheets of tumor cells with pale cytoplasm due to marked glycogen accumulation, alongside abundant interstitial collagen. They have a predilection for spinal localization as well as for the cerebellopontine angle. Due to their biological behavior, which is characterized by a high recurrence rate, CCMs are classified as WHO tumor grade 2 [83]. The molecular function of SMARCE1 is highly diverse; the molecular cascade resulting from a *SMARCE1*-mutation in CCM remains speculative at this time, as a wide variety of different mutations (frameshift, nonsense, deletions, insertions) have been described [83,97]. The complexity of determining the exact cascade of tumorigenesis resulting from a SMARCE1 mutation is evident from the fact that, in SMARCE1-knockout cells, a total of 1684 genes were downregulated and 1772 genes were upregulated [98]. The primary physiological function of SMARCE1 is believed to be its involvement in stabilizing the entire canonical BAF complex (cBAF) and its central ATPases, whereas it has no effect on PBAF [98]. An in vitro study of SMARCE1 knockout cells demonstrated a reduction in the chromatin-binding activity of cBAF and an apparently reactive activation of the non-canonical complex ncBAF, accompanied by upregulation of tyrosine kinase pathways, the WNT signaling pathway, and other pathways. The maintenance of an oncogenic program resulting from the activation of ncBAF is therefore suspected to be a central consequence of the loss of SMARCE1 [97,98].

**Figure 2:**
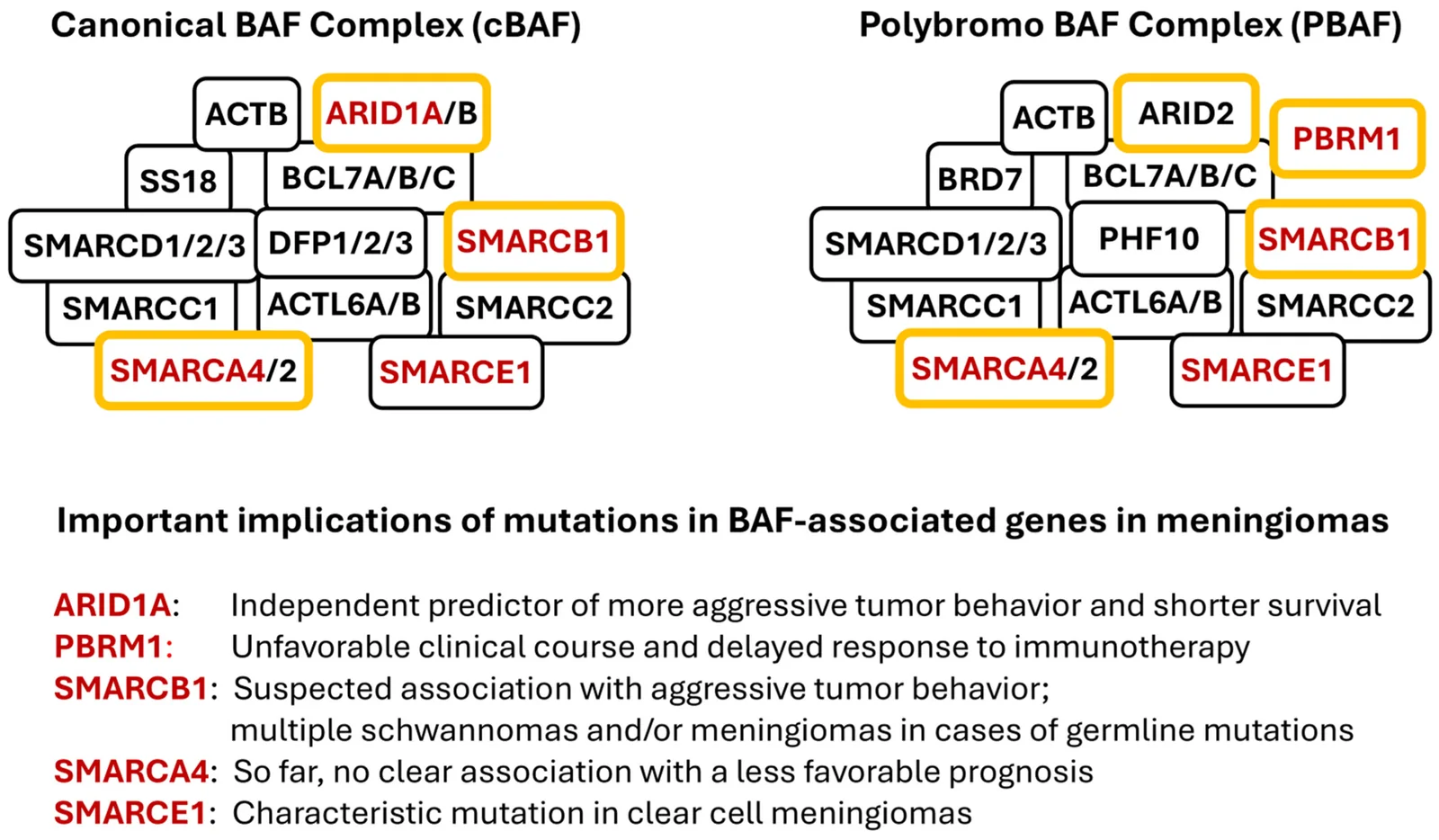
Schematic representation of the canonical BAF complex (cBAF) and the polybromo BAF complex (PBAF), each showing all components. Orange Frame: the BAF-associated components most frequently mutated in malignant tumors according to a pan-cancer study; Red Letters: the BAF-associated components most frequently mutated in meningiomas, along with the most significant implications of these mutations as described in the text [74,75,78,79,84,86,88,92,94,95,96]. **Abbreviations in alphabetical order**: ACTB: actin beta; ACTL6A/B: actin-like 6A/B; ARID1A/B: AT-rich interaction domain 1A/B; ARID2: AT-rich interaction domain 2; BCL7A/B/C: BAF chromatin remodeling complex subunit 7A/B/C; BRD7: bromodomain containing 7; DFP1/2/3: double PHD fingers 1/2/3; PBRM1: polybromo 1; PHF10: PHD finger protein 10; SMARCA2/A4: SWI/SNF-related matrix-associated actin-dependent regulator of chromatin A2/4; SMARCB1/C1/C2/D1/D2/D3/E1: SWI/SNF-related matrix-associated actin-dependent regulator of chromatin B1/C1/C2/D1/D2/D3/E1; SS18: SS18 subunit of BAF chromatin remodeling complex.

All these alterations in components of the BAF complexes described in meningiomas are summarized graphically in Figure 2. Another challenge associated with SWI/SNF complexes in general is that the influence of non-coding RNA molecules on SWI/SNF complexes, as well as the role of other chromatin remodeling complexes such as ISWI, CHD, and INO80, remains largely unknown in many types of tumors, particularly meningiomas. The significance of these aspects will be addressed in the discussion section below.

## 5. Non-Coding RNA Molecules

### 5.1. Micro RNAs (miRNAs)

Micro-RNAs (miRNAs) are short, non-coding RNA molecules ranging in length from 19 to 25 nucleotides. They are derived from pre-microRNA sequences of approximately 70 nucleotides in length [99,100]. Key enzymes in miRNA biogenesis are the Type III RNAse DROSHA and the microprocessor-complex DGCR8 (Di-George syndrome critical region gene 8), which are involved in the formation of pre-miRNAs in the cell nucleus. The transport protein exportin-5 transports the pre-miRNA into the cytoplasm, where it is cleaved into the final miRNA by the endoribonuclease DICER. The RISC protein complex (RNA-induced silencing complex) is the key protein complex involved in regulating the interaction between miRNA and mRNA [101,102]. It is emphasized that many details of miRNA biogenesis and functionality remain poorly understood to this day, particularly regarding the structure and dynamics of the RISC multiprotein complex [101]. Micro-RNAs exert their effects at the post-transcriptional level by binding to the 3’-end of the untranslated region of a messenger RNA (mRNA). This binding usually results in reduced translation and consequently reduced protein expression, but several miRNAs can also lead to increased translation and enhanced expression [99,100]. Of note, individual miRNAs may influence the expression of multiple genes [100]. A distinctive feature of many miRNAs is that they can be detected in peripheral serum. Consequently, oncological studies on miRNAs have been conducted partly using tumor tissue and partly through analysis of peripheral serum [103].

Of all types of non-coding RNA molecules, miRNAs are by far the most extensively studied in tumors. The results are valid only for the specific tumor type under investigation, but there are individual miRNAs that act as tumor suppressors and are downregulated in many types of tumors, such as miRNA-145 [100,104]. Similarly, there are oncogenic miRNAs like miRNA-21, which are overexpressed in several tumor types [105,106]. In meningiomas, a comparison of tumors from 83 patients with 8 normal brain tissue controls revealed increased expression of the miRNAs 574, 26b, 335, and 98 in the tumors [107]. Another research group found a downregulation of miRNA-195, which leads to the overexpression of its target, fatty acid synthase (FASN) [108]. FASN catalyses the synthesis of the fatty acid palmitate, whose incorporation into cysteine residues of proteins (palmitoylation) can lead to altered activity of multiple cancer immunology-related membrane proteins in tumors, such as PD-L1 [109,110]. FASN is overexpressed in many tumor types, such as colorectal and hepatocellular carcinoma (HCC), and contributes significantly to tumor proliferation [110,111]. The exact mechanism of action is still being investigated; experimental evidence in HCC suggests increased tumor cell death due to enhanced activity of CD8+ cells following downregulation of FASN [110]. It is important to note that these findings cannot simply be generalized to different tumor types. In the case of meningiomas, it was shown that experimental overexpression of miR-195 had a stronger antiproliferative effect than downregulation of FASN. Therefore, the authors place greater emphasis on the miRNA itself and highlight that further studies are necessary to identify additional targets of miR-195 [108]. It should also be noted that overexpression of certain tumor-suppressive miRNAs has also been observed in meningiomas. One example is miRNA-218, which was overexpressed in meningiomas from 15 patients compared to control tissue. It is believed that this miRNA plays a role in preventing or slowing the malignant progression of grade I and II meningiomas [112]. This finding of miRNA-218 overexpression in meningiomas could not be confirmed in other tumor types, particularly in malignant tumors, where downregulation of this miRNA was observed in each case. Under physiological conditions, miRNA-218 has an inhibitory effect on numerous targets such as the epidermal growth factor receptor (EGFR) or the phosphatidylinositol 3-kinase and protein kinase B pathway (PI3K/AKT), thereby inhibiting the proliferation of malignancies. The differing expression patterns of miRNA-218 in malignant tumors and, in contrast, in predominantly benign meningiomas require further investigation [112,113,114]. Oncogenic miRNAs are also overexpressed in meningiomas; this applies to miRNAs 21, 335, 224, 29c, and 483. Individual oncogenic miRNAs inhibit different tumor suppressors such as TP53 and PTEN (miRNA-21), RB1-protein (miRNA-335), and PTX3 (miRNA-29c) leading to enhanced proliferation [105,106,115,116,117]. Other oncogenic miRNAs can lead to the activation of genes with tumor-promoting effects, as in the case of IGF2 activation by miRNA-483 [118]. For the oncogenic miRNA-224, a particularly strong correlation was observed between its increased expression level and the tumor grade of meningiomas, as well as an inverse correlation with the duration of the recurrence-free interval. Downregulation of the two targets of miRNA-224, ERK2 (extracellular signal-regulated kinase 2) and BAK (BCL-2 homologous antagonist/killer), leads to an inhibition of tumor cell apoptosis [116,119]. To date, not all miRNAs have been extensively studied with regard to their targets, including their precise molecular behavior in meningiomas. The miRNAs for which there is strong evidence of an influence on specific targets and signaling pathways, as well as those miRNAs showing significant changes in expression compared to control cases and across different tumor grades, are listed in Table 2.

**Table 2:** Dysregulated micro-RNAs (miRNAs) in meningiomas due to their increased (↑) or decreased (↓) expression; target(s) of the miRNA; findings on the significance of these miRNAs in meningiomas.

Micro RNA/Reference(s)	Important Target(s)	Findings on the Significance of These miRNAs in Meningiomas
miRNA-195 ↓ [108]	FASN	Tumor suppressor; downregulated in meningiomas with higher tumor grade leading to increased expression of FASN and enhancement of proliferation
miRNA-34a-3p ↓ [120]	SMAD4, FRAT1, BCL2	Downregulation of this miRNA in meningiomas led to increased proliferation and inhibited apoptosis of tumor cells
miRNA-451 ↑ [121,122]	HMGB1, RAB14, NF-κB pathway	Although this miRNA is overexpressed in meningiomas, it is considered a tumor suppressor in many tumors; there is no difference in its expression across different tumor grades of meningiomas
miRNA-497 ↓ + miRNA-219 ↓ [123,124]	CCND1	Tumor suppressor-miRNAs; expression decreases with higher tumor grade; the combined detection of both miRNAs in peripheral serum could differentiate between meningioma patients and controls
miRNA-21 ↑ [105,106]	P53, PTEN, BCL2, NF-κB pathway	Oncogenic miRNA in different tumor types; wide range of targets with downregulation of numerous tumor suppressors; higher expression in grade II and III meningiomas compared to grade I
miRNA-218 ↑ [112,113]	EGFR, Survivin, PI3K/AKT pathway	Tumor suppressor; it is hypothesized that this upregulation plays a role in delaying the malignant transformation of low-grade meningiomas
miRNA-335 ↑ [115,116]	RB1 protein	Oncogenic miRNA; its overexpression correlates with the grade of meningiomas; because it directly targets RB1, this is considered to be the cause of enhanced cell growth and impaired cell-cycle arrest
miRNA-145 ↓ [100,104,116]	COL5A1, IGF1, FSCN1	Tumor suppressor with reduced expression in many tumor types; downregulated in meningiomas grade II and III, in contrast to low grade meningiomas
miRNA-224 ↑ [116,119]	ERK2, BAK	Oncogenic miRNA; in meningiomas, its expression correlates with tumor grade and inversely with the duration of recurrence-free survival
miRNA-107 ↓ [125,126]	EGFR, CPEB3,	Tumor suppressor in meningiomas; the degree of downregulation correlates with tumor grade; a striking contrast to other tumors such as HCC, in which miRNA-107 promotes proliferation
miRNA-29c-3p ↑ [117]	PTX3	Oncogenic RNA; appears to play a role in enhancing tumor-promoting inflammation in meningiomas by targeting PTX3; inhibition of apoptosis
miRNA-193b-3p ↓ [127]	CCND1	Tumor suppressor; downregulation of this miRNA leads to increased expression of cyclin D1 and enhanced proliferation in grade II and III meningiomas compared to grade I meningiomas
miRNA-26a-5p ↓ + miRNA-101-3p ↓ [128]	CKS2	Tumor suppressors; downregulation of both mi-RNAs leads to increased expression of the cell cycle regulator CKS2 and enhanced proliferation of meningioma cells in vitro; CKS2expression was significantly higher in higher tumor grades
miRNA-15a ↓ + miRNA-146a-5p ↓ + miRNA-331-3p ↓ [129]	NF-κB pathway	Tumor suppressors; three downregulated miRNAs with strong correlation with time to recurrence in a large cohort of meningioma patients; the NF-κB pathway is the primary target of the first two miRNAs, the target of miRNA-331-3p in meningiomas remains unknown
miRNA-16 ↓ + miRNA-519 ↓ [130]	HuR	Tumor suppressors; due to reduced expression of both miRNAs in meningiomas, inhibition of the proliferation-promoting RNA-binding protein HuR is also reduced
miRNA-483-5p ↑ [118]	IGF2	Oncogenic miRNA; increased expression of this miRNA in meningiomas leads to enhanced activation of its target, IGF2, and to increased proliferation of tumor cells

**Abbreviations of the targets in alphabetical order:** BAK: BCL-2 homologous antagonist/killer; BCL2: B-cell lymphoma 2 apoptosis regulator; CCND1: Cyclin-D1; CKS2: CDC28 protein kinase regulatory subunit 2; COL5A1: collagen alpha-1(V) chain; CPEB3: cytoplasmic polyadenylation element binding protein 3; EGFR: epidermal growth factor receptor; ERK2: extracellular signal-regulated kinase 2; FASN: fatty acid synthase; FRAT1: frequently rearranged in advanced T-cell lymphomas; FSCN1: fascin actin-bundling protein 1; HMGB1: high mobility group box 1; HuR: human antigen R; IGF1: insulin-like growth factor 1; IGF2: insulin-like growth factor 2; JAM3: junctional adhesion molecule 3; NF-κB: nuclear factor kappa-light-chain-enhancer of activated B cells; P53: tumor protein p53; PI3K/AKT: phosphatidylinositol 3-kinase (PI3K) and protein kinase B (AKT); PTEN: phosphatase and tensin homolog; PTX3: pentraxin 3; RAB14: ras-related protein Rab-14; RB1: retinoblastoma transcriptional corepressor 1; SMAD4: SMAD Family Member 4; Survivin: baculoviral IAP repeat containing 5.

### 5.2. Long Non-Coding RNAs (lncRNAs)

By definition, non-coding RNA molecules longer than 200 nucleotides are referred to as long non-coding RNA molecules (lncRNAs). These can be transcribed in various chromosomal regions, including introns and repetitive sequences; transcription by all eukaryotic RNA polymerases I-III or their precursors has been described in humans [131,132]. LncRNAs are involved in various cellular processes, such as cellular differentiation, proliferation, and other physiological processes. The targets of lncRNAs are often microRNAs (miRNAs), but miRNA-independent functions of individual lncRNAs have also been demonstrated [133]. Depending on the function and integration of lncRNAs into specific signaling pathways, their effects can either promote or inhibit cellular growth and proliferation, as well as apoptosis. In contrast to the research on the function of miRNAs, there is comparatively less knowledge regarding lncRNAs in meningiomas. Significant alterations in the expression of individual lncRNAs have been reported in meningiomas, although the exact molecular mechanism underlying this dysregulation remains largely unexplained. For some of these lncRNAs, there is evidence that a specific miRNA acts as a target molecule, as well as evidence of specific signaling pathways that are influenced by changes in the expression of the respective lncRNA in meningiomas. LncRNA MIR17HG promotes tumor growth and is overexpressed in many tumors such as osteosarcoma, cervical squamous cell carcinoma, colorectal cancer, and gastric cancer, as well as in CNS tumors such as gliomas and atypical teratoid rhabdoid tumors. Elevated expression is also known in WHO Grade I and II meningiomas; however, unlike the signaling pathways of this lncRNA in other tumor types that have already been described, those in meningiomas remain unknown to date [134,135]. Another lncRNA called anti-differentiation ncRNA (ANCR) is also elevated in various tumors. When comparing different types of brain tumors, higher expression was observed in pituitary adenomas and gliomas, whereas significantly lower expression was observed in meningiomas [136]. Additional in vitro investigations of the same study group have demonstrated that ANCR has an anti-apoptotic effect and promotes cell proliferation [136]. Reduced expression of the lncRNAs “Metastasis-associated lung adenocarcinoma transcript 1” (MALAT1) and “telomeric repeat-containing RNA” (TERRA) was observed in meningiomas compared to control cases [137,138]. MALAT1 is overexpressed in various malignancies; in meningiomas, however, its expression inversely correlates with the aggressiveness of the tumor. The cause of this correlation and the contrasting expression pattern compared to other malignancies remains unclear; a correlation with the expression of cell cycle regulators such as E-cadherin or cyclin D1 could not be demonstrated in meningiomas [137]. TERRA is expressed at the telomeric ends of most human chromosomes and plays a role in maintaining telomere length and chromosomal stability. Following expression, there is a free form (f-TERRA) and a hybridized form (h-TERRA), which exert their effects on the chromosome and the telomere. Pre- and postoperatively, the concentration of f-TERRA in peripheral blood was significantly reduced in patients with meningiomas, whereas telomere length in meningioma patients was significantly increased. It is hypothesized that increased telomere length may promote the development of meningiomas. Nevertheless, it is noted that the precise telomere dynamics and the involvement of the lncRNA TERRA in meningiomas require further investigation [138].

**Figure 3:**
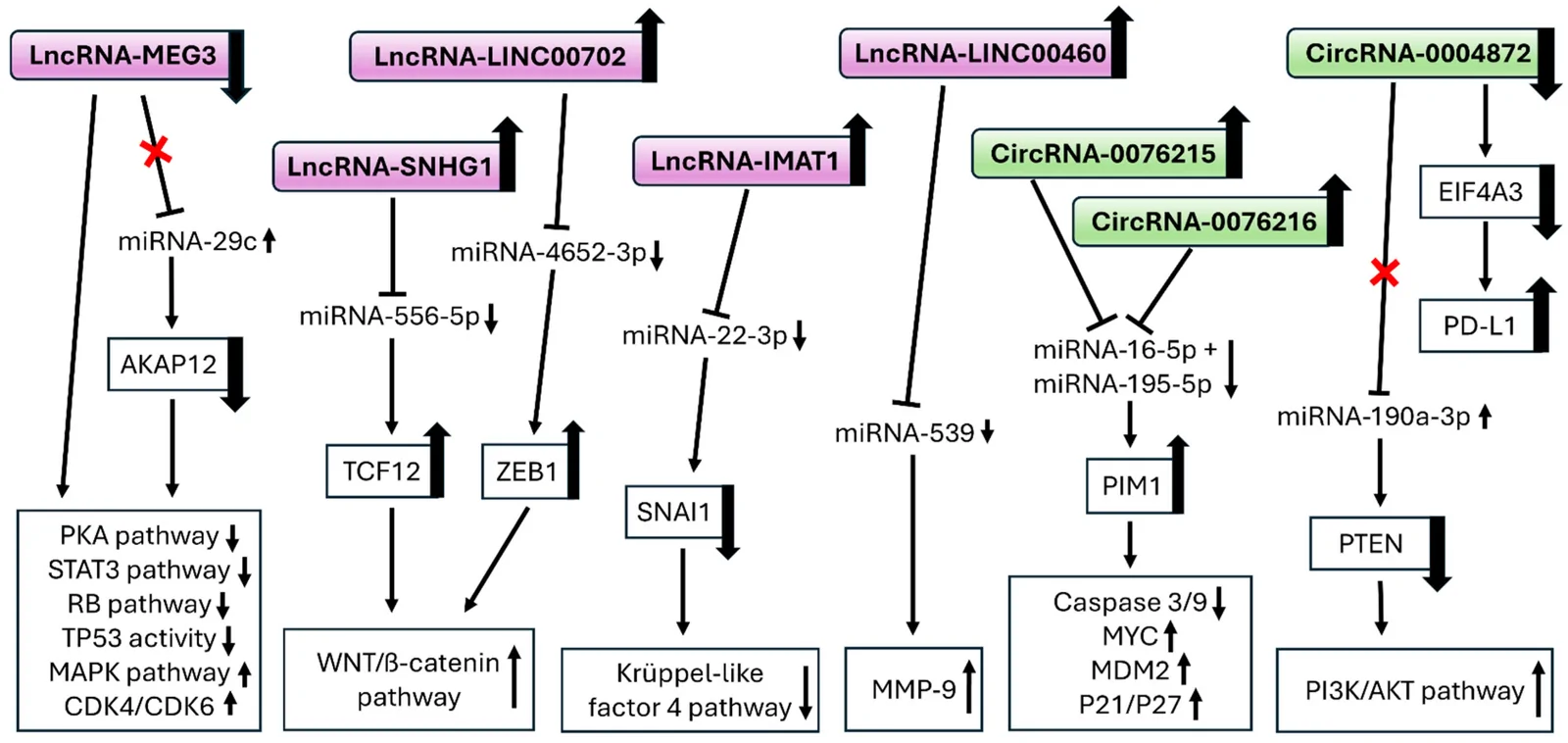
Dysregulated long non-coding RNAs (lncRNAs) and circular RNAs (circRNAs) in meningiomas due to their increased (↑) or decreased (↓) expression, leading to downregulation (↓) or upregulation (↑) of microRNAs (miRNAs), proteins, caspases, and signaling pathways, as described in the text. Red: lncRNAs; Green: circRNAs; Red Cross: inhibited blocking of a miRNA due to downregulation of a lncRNA or a circRNAs [99,139,140,141,142,143,144,145,146,147,148,149,150].

There is already strong evidence regarding the influence of several lncRNAs on specific signaling pathways and miRNAs in meningiomas. LncRNA-MEG3 (maternally expressed gene 3) is downregulated in meningiomas along with the tumor suppressor AKAP12 (A-kinase anchor protein 12) [139]. Physiologically, LncRNA-MEG3 acts as a sponge and thus as an inhibitor of miRNA-29c, which in turn leads to increased expression of AKAP12. Conversely, reduced MEG3 expression in meningiomas leads to inhibited blocking of miRNA-29c and reduced expression of AKAP12, as confirmed in vitro [140,141]. The two tumor suppressors lncRNA-MEG3 and AKAP12 influence numerous signaling pathways. When their expression is reduced, this promotes tumor growth, for example, as a result of hypophosphorylation of tumor protein 53 (TP53), increased expression of cyclin-dependent kinases 4 and 6 (CDK4, CDK6), as well as inhibition of the retinoblastoma (RB) pathway [140,141]. It was also reported that two overexpressed lncRNAs activate the Wingless-int-1/β-catenin signaling pathway (WNT/β-catenin pathway). LncRNA-SNHG1 (small nucleolar RNA host gene 1) acts as an inhibitory sponge for miRNA-556-5p, which, in the case of SNHG1 overexpression, leads to the activation of TCF12 (transcription factor 12) and thus to the activation of the WNT/β-catenin pathway [99]. Similarly, the overexpressed lncRNA LINC00702 inhibits miRNA-4652-3p, which leads to the activation of the transcription factor ZEB1 (Zinc Finger E-Box Binding Homeobox 1) and subsequently of the WNT/β-catenin pathway [142]. The lncRNA IMAT1 (Invasive meningioma-associated transcript 1), which is overexpressed in meningiomas, leads to the inhibition of miRNA-22-3p and, consequently, to the inhibition of the transcriptional repressor SNAIL1 (Snail Family Transcriptional Repressor 1). This, in turn, inhibits the tumor-suppressive activity of the entire Krüppel-like factor 4 (KLF4) pathway [143]. Inhibition of miRNA-539 by the lncRNA LINC00460, in turn, promotes proliferation in meningiomas due to increased expression of matrix metalloproteinase 9 (MMP-9) (Figure 3) [144]. A comparison of meningioma patients with and without recurrence confirmed that the lncRNAs studied to date represent only a small fraction of the total number likely to be involved in meningiomas. The study demonstrated significantly different expression levels in 108 lncRNAs, with statistically higher expression of the three lncRNAs GOLGA6A-1, ISLR2, and AMH in cases with recurrence. The authors consider GOLGA61-1 in particular to be a reliable predictor of meningioma recurrence, but note that further research is needed on the molecular mechanisms underlying these lncRNAs [145].

### 5.3. Other Types of Non-Coding RNAs

Among the various types of non-coding RNAs, altered expression patterns have also been detected in meningiomas for circular RNAs (circRNAs) and small nucleolar RNAs (snoRNAs). CircRNAs are formed by back-splicing of the precursor mRNA and are characterized by a covalent bond between a downstream 3′ end of the RNA and a 5′ start site that is normally located upstream [146]. Their targets are mostly miRNAs, which are inhibited by the corresponding circRNA. Overexpression of the two circRNAs 0076215 and 0076216 in meningiomas leads to the inhibition of the two tumor-suppressor microRNAs miRNA-15-5p and miRNA-195-5p, thereby activating the proto-oncogene Pim1-proto-oncogene/serine/threonine kinase (PIM1). PIM1, in turn, promotes the inhibition of apoptosis through reduced expression of caspases 3 and 9, as well as increased expression of the cell cycle regulators p21 and p27, the MYC proto-oncogene (MYC) and the MDM2 proto-oncogene (MDM2). Therefore, the authors discuss the potential therapeutic benefits of PIM1 inhibitors [147,148,149]. CircRNA-0004872 is downregulated in meningiomas; a positive correlation has been observed between its expression and good relapse-free survival in meningioma patients. As a result of the reduced expression, the inhibitory effect on miRNA-190-3p is blocked, leading to reduced expression of Phosphatase and Tensin Homolog (PTEN) and activation of the phosphoinositide 3-kinase and protein kinase 2/AKT (PI3K/AKT) pathway (Figure 3). Another tumor-promoting effect of the reduced expression of circRNA-0004872 is the downregulation of eukaryotic translation initiation factor 4A3 (EIF4A3), which in turn promotes immune evasion through upregulation of programmed death-ligand 1 (PD-L1). Overall, circRNA-0004872 is considered as a tumor-suppressor with potential therapeutic significance in meningiomas [148,150].

Small nucleolar RNAs (snoRNAs) are involved in the modification of ribosomal RNA; dysregulated expression of individual snoRNAs has been detected in various tumors. Their mechanism of action is complex, as a single snoRNA can bind to multiple RNA segments, and multiple snoRNAs can target the same binding site [151]. In an earlier study from 2002, reduced expression of the snoRNA h5sn2 was detected in meningiomas, while the snoRNAs h5sn1, h5sn3, and h5sn4 showed no altered expression compared to normal brain tissue. This finding was not further investigated in the subsequent period [152]. A comparable result was observed for snoRNAs 46 and 48, whose expression is significantly reduced in high-grade meningiomas with tumor grades II and III but only slightly reduced in meningiomas with tumor grade I. These snoRNAs studied to date in meningiomas are therefore considered tumor suppressors, although the authors have noted that their exact function remains unclear [153].

## 6. Discussion

The genetics of meningiomas have been studied for many years. The most common genetic abnormality is a mutation in the neurofibromatosis type 2 (NF2) gene, which occurs as part of the NF2 tumor predisposition syndrome but is also found in the majority (60%) of sporadic meningiomas [154]. In meningiomas without an NF2 alteration, other oncogenic mutations can be detected, such as mutations of the genes for AKT serine/threonine kinase 1 (AKT1), TNF receptor-associated factor 7 (TRAF7), KLF transcription factor 4 (KLF4), smoothened frizzled class receptor (SMO), or phosphatidylinositol bisphosphate 3-kinase catalytic subunit alpha (PIK3CA) [154,155]. Histopathological examination and tumor grading continue to be based on the classic criteria such as cellularity, nucleus-to-cytoplasm ratios, and mitotic count. In addition, genetic criteria are also taken into account. In particular, the detection of a homozygous deletion of *cyclin dependent kinase inhibitor 2A/B (CDKN2A/B)* and/or a mutation of *telomerase reverse transcriptase (TERT)* in a meningioma directly leads to the assignment of WHO tumor grade 3 [22,155,156]. Furthermore, meningiomas with a higher tumor grade may exhibit a variety of additional genetic aberrations, such as copy number variations including losses of 1p, 10q, or 14q [19,155,156].

The possibility of performing methylation analysis is mentioned in the latest WHO classification of CNS tumors; since its publication, diagnostic and prognostic significance of DNA methylation in meningiomas has become increasingly evident [155]. The most important finding is that methylome analysis reveals clear prognostic differences between the various clusters, differences that are not necessarily reflected by the tumor grade of the individual case. This may particularly apply to certain cases diagnosed histologically as atypical meningiomas with tumor grade 2, in which, based on additional analysis of copy number variations (CNVs) and methylation profiling, a different biological behavior is expected than that indicated by the tumor grade. The same may apply to meningiomas with tumor grade I, in which a more aggressive behavior is expected from a genetic and epigenetic perspective. For these cases with divergent prognostic assessments based on histopathology and molecular biology, it is recommended to add the note “with molecular data suggestive of lower (higher) risk of recurrence” after the histopathological diagnosis and tumor grade have been specified [22]. With regard to the reproducibility of methylation-based classification methods, all working groups have now evaluated a large number of meningioma cases; the reported case numbers range up to an international cohort of 1698 cases [2] and 3031 cases in another working group [21]. The results were validated in each case using validation cohorts [2,3,21]. Standardization of the array analysis method, which remains the predominant approach, including the necessary sample preparation, has also been established. Sample purity for analysis is very high in meningiomas due to the predominantly solid tumor compartment. Only in the relatively rare lymphoplasmacyte-rich meningiomas and angiomatous meningiomas, a higher proportion of non-neoplastic components has been reported [22]. The independent prognostic value of methylome analysis was also demonstrated in one of the studies by the nearly complete overlap of group-specific patient outcomes. This overlap was confirmed by comparing the statistical analysis based exclusively on methylome analysis data with the analysis that took into account both methylome analysis and genetic analysis data [2]. In addition to the classification of meningiomas using methylation analysis, studies involving the analysis of individual genes are also worth mentioning, as the specific significance of these genes is not automatically apparent in the aforementioned classification studies (Table 1a,b). Of particular note is the hypermethylation and consequent inactivation of the tumor suppressor *WNK2*, which occurs frequently in meningiomas but only in isolated cases of other tumor types, and thus appears to represent a specific epigenetic aberration in meningiomas [23]. Also noteworthy is the detection of hypomethylation of *FOXC1* and its target *FOXCUT*, each in a paired comparison with normal dura mater tissue. This observation calls for further scientific investigation, especially since research into the genetic and epigenetic changes that play a role in the development of meningiomas from their tissue of origin is still in its infancy [29].

Regardless of the disease or tumor type under investigation—in this case, meningiomas—other forms of methylation with oncological relevance must be mentioned, as the previously held view that 5-methyl-cytosine (5mC) is the only methylation product of the human DNA is no longer valid today [157]. Hydroxymethylation of cytosine (5hmC) occurs through the oxidation of 5mC by ten-eleven translocation methyl-cytosine dioxygenases (TETs), whereby a hydroxyl group replaces a hydrogen atom at the 5’ end of the methyl-cytosine. Kriaucionis and Heintz first discovered this form of methylation in human Purkinje cells in 2009 [158,159,160]. A significantly reduced level of 5hmC has been detected in various malignancies, such as melanoma, hepatocellular carcinoma, breast cancer, bladder cancer, and glioblastomas. It has been shown that the extent of 5hmC reduction correlates with a poorer prognosis in some tumor types [161,162,163,164]. For meningiomas, there are currently no findings regarding the occurrence of hydroxymethylation. The same applies to two other forms of methylation, namely adenine methylation (6mA) and non-CpG methylation, also known as “CpH methylation”, where “H” can be represented by the other bases except guanine (G), i.e., adenine, thymine, or cytosine. Methylation of adenine at position 6 (6mA) is significantly reduced in various types of tumors, such as gastric and hepatocellular carcinoma [165,166,167,168]. CpH-methylation has been reported in humans in pluripotent stem cells, cortical neurons, and tumor cells [169,170,171]. A higher proportion of CpH-methylated cytosines was detected in the mitochondrial DNA (miDNA) of tumor cells from hepatocellular carcinoma compared with normal liver cells [172]. Overall, the current findings on these additional forms of DNA methylation underscore their relevance for tumor research in general and, consequently, for further methylome studies on meningiomas.

Regarding histone modifications in meningiomas, current evidence suggests that the loss of lysine 27 trimethylation in H3 (H3K27me3) has the greatest prognostic significance. This alteration correlates with a more aggressive tumor behavior [35,36,37,38,39]. In addition to this prognostically significant form of histone modification, two other forms have been described on histone 3 (H3), each of which is associated with more aggressive tumor behavior: trimethylation of lysine at position 3 (H3K4me3) and di- or trimethylation of lysine at position 9 (H3K9me2/3). These two histone modifications have been detected in individual meningiomas; further studies on meningiomas will have to determine whether they have a similarly significant prognostic value as the loss of H3K27me3 [9,33,45,47]. The significance of histone acetylation in meningiomas is primarily evident in the tumor-inhibiting effect of HDAC inhibitors (HDACis), which has been demonstrated in several experimental and in vitro studies [37,50,51,53]. A particularly encouraging observation is the enhanced effect of oncolytic HSV therapy following the additional administration of the pan-HDAC inhibitor (HDACi) panobinostat [52]. It is considered necessary to further investigate the effect and thus the potential application of HDACis in various molecular subgroups, as one study showed that the HDACi vorinostat had different effects across different molecular meningioma groups [37]. Histone acetyltransferases (HATs) have been relatively less studied compared to HDACs, and increased expression of HAT1 has been demonstrated in meningiomas with a higher tumor grade. A systematic investigation of the prognostic and potential therapeutic significance of HAT1 in a large cohort of meningioma cases is considered necessary [57]. The same applies to the histone modification known as phosphorylation of histone H3 at serine 10 or serine 28 (PHH3), which is not a pathological modification but merely a reliable marker of mitosis. Findings to date in meningiomas confirm a significant correlation between this marker and the numerically determined mitosis count, as well as with prognosis. It should be noted that very little is currently known about the molecular mechanism underlying this histone modification [59,60].

Nothing is yet known about other forms of histone modification in meningiomas; however, their oncological relevance and their occurrence in a wide variety of tumor types have been conclusively demonstrated. Small ubiquitin-like modifiers (SUMOs) are a class of five different proteins, their covalent binding to lysine residues on target proteins, such as histones, is referred to as SUMOylation [173]. In glioblastomas, increased activity of metabolic and proliferative signaling pathways has been observed as a result of SUMOylation, which is therefore considered a potential therapeutic target for this tumor entity [174]. Arginine can be hydrolysed in histones by peptidyl-arginine deiminases (PADs), a process known as citrullination [175,176]. Overexpression of various members of the PAD family has been reported in many types of malignant tumors, such as breast cancer, hepatocellular carcinoma, and pancreatic carcinoma. It is hypothesized that PAD-induced histone citrullination directly stimulates tumor cell proliferation and promotes a tumor-favouring immune environment [34,177]. Histone lactylation occurs through the covalent binding of lactate to lysine. This histone modification occurs on various histone proteins and promotes the progression of malignant tumors, such as glioblastomas due to the lactylation of H4K8 or gastric carcinoma due to the lactylation of H3K18 [178,179]. Histone crotonylation is primarily a physiological process in which the metabolite crotonyl-CoA adds a crotonyl group to lysine residues on histones, contributing to a variety of processes, such as the maturation of germ cells and neurons [180]. In oncology, depending on the tumor type, both the addition and removal of crotonyl groups from histones can contribute to tumor proliferation, as confirmed by in vitro studies on breast cancer and glioblastoma cells [181,182]. To date, no study has been conducted on these four oncologically relevant forms of histone modification (SUMOylation, citrullination, lactylation, and crotonylation) in meningiomas.

Although known for a long time, chromatin-remodeling complexes remain the subject of intensive research because their exact mechanisms of action have not yet been fully elucidated. In meningiomas, mutations have been detected in components of the SWI/SNF complexes cBAF and PBAF, namely ARID1A, PBRM1, SMARCA4, SMARCB1, and SMARCE1 [75,83,84,85,90]. It is important to note that the mutated proteins are merely components of larger complexes. It therefore seems appropriate to also consider investigating those chromatin-remodeling complexes for which no explicit findings regarding meningiomas have yet been reported. These include the non-canonical SWI/SNF complex (ncBAF), the imitation switch complex (ISWI), the chromodomain-helicase DNA-binding protein complex (CHD), and the inositol-requiring mutant 80 complex (INO80). ncBAF is a complex that is predominantly localized in the regions of CTCT sites (insulators), and its specific components exhibit dysregulation due to under- or overexpression in various tumor types [70,71]. In contrast to the cBAF and PBAF complexes, which tend to act as tumor suppressors, ncBAF frequently exhibits oncogenic potential, leading to the activation of oncogenes and cellular proliferation [70]. The complexes ISWI and CHD perform various functions related to the interplay between chromatin and histones, such as the formation of prenucleosomes and the subsequent generation of evenly spaced nucleosomes [183,184]. In contrast, the INO80 complex is capable of altering the architecture of the nucleosome by replacing histones, thereby enhancing or blocking gene expression [87,183,184]. Just as for ncBAF, dysregulation of ISWI, CHD, and INO80 has also been described in various types of malignant tumors. The specific changes vary among the different tumor types and are mostly associated with mutations and overactivity of the respective complex [184,185]. Descriptions of corresponding alterations of cBAF, ISWI, CHD, or INO80 are currently lacking for meningiomas.

Concerning the topic of non-coding RNAs, several studies on microRNAs that demonstrate dysregulation in meningiomas are particularly noteworthy. Of special note are miRNAs such as miRNA-224, whose expression correlates with tumor grade and the likelihood of tumor recurrence [184,185]. The potential significance of miRNAs as biomarkers in peripheral serum should also be emphasized. For example, significantly reduced serum levels of miRNA-497 and miRNA-219 were demonstrated in meningioma patients compared to control cases, as well as when comparing cases with tumor grades 2 and 3 to those with tumor grade 1 [123,124]. Several studies on meningiomas have also demonstrated that an increased or decreased expression of long non-coding RNAs and circular RNAs has a significant impact on specific signaling pathways, ultimately promoting tumor growth. General challenges in studies of non-coding RNAs (ncRNAs) include the often low expression levels in individual cases under investigation, as well as the identification of specific targets, such as certain miRNAs as targets for an lnRNA [131]. In contrast to methylome analysis, which is already used for diagnostic purposes, the aforementioned studies on ncRNAs are predominantly comparative studies between tumors of different grades and control cases; diagnostic implementation is currently only a prospect. To further optimize reproducibility, there is a call for further standardization of in vitro assays, as well as validation of the existing results in additional, larger cohorts [130].

**Figure 4:**
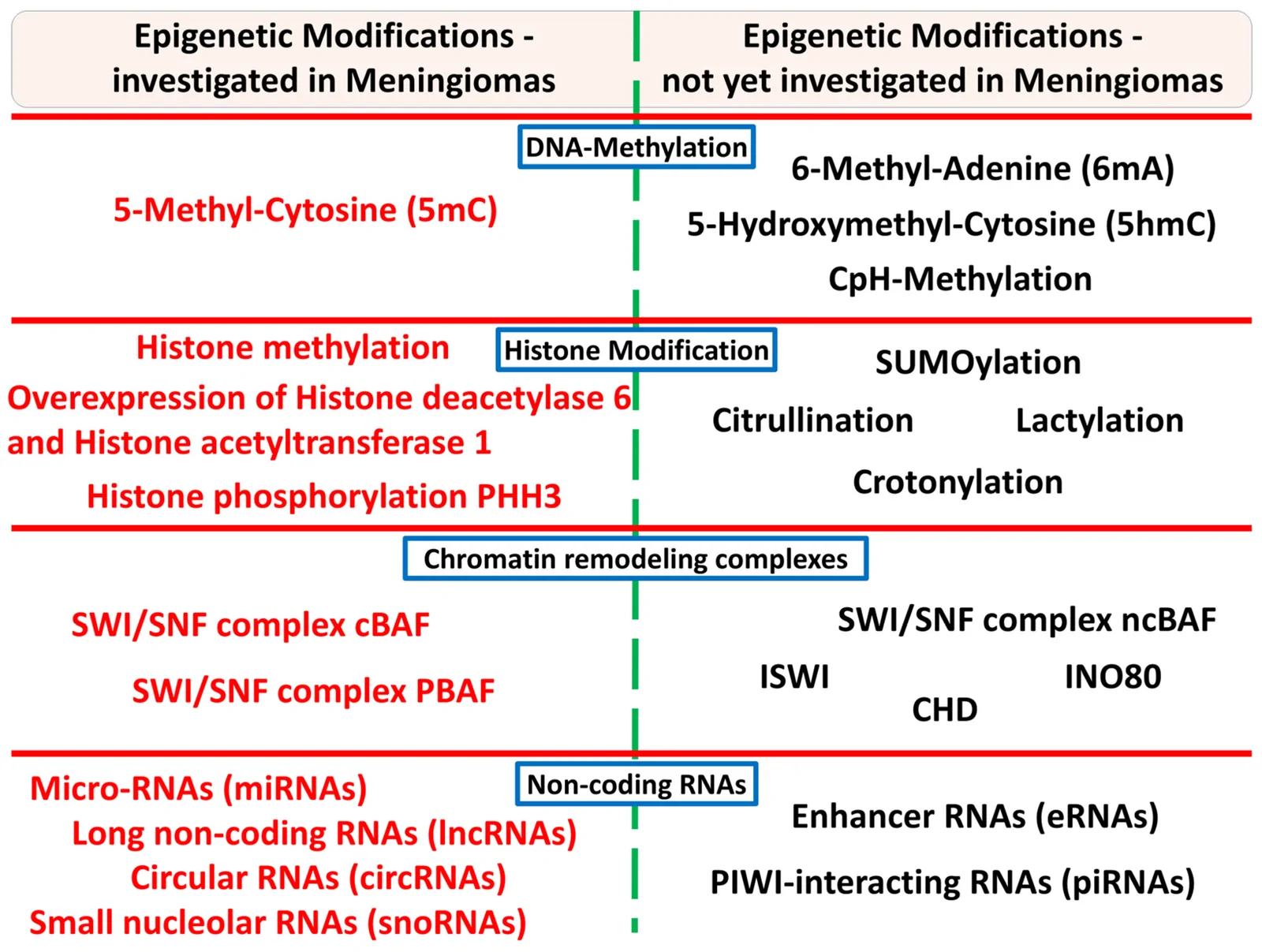
Epigenetic modifications investigated and not yet investigated in meningiomas as described in the text [53,56,59,60,70,71,159,160,161,162,163,167,169,174,176,179,181,183,184,185,186,187,188,189,190,191,192,193,194,195].

There is currently no insight into the significance of two further oncologically relevant forms of ncRNAs in meningiomas, namely enhancer RNAs (eRNAs) and P-element-induced wimpy testis (PIWI) interacting RNAs (piRNAs). eRNAs are encoded in enhancer regions and are involved in transcriptional regulation. In tumors such as breast cancer or prostate cancer, they often exhibit aberrant expression and are involved in the activation of oncogenes [186,187,188]. piRNAs were originally described in Drosophila flies and represent a key gene for gametogenesis in various species. The name is derived from the failure of germline stem cell maintenance in gonads of PIWI mutants [189,190,191]. Their dysregulation in a wide variety of tumor types is well established. The expression of specific piRNAs such as piRNA-8041 is downregulated in advanced-stage glioblastomas, whereas other piRNAs such as piRNA-651 are overexpressed in other tumor types, such as non-small-cell lung cancer [192,193,194,195]. Further research into this category of epigenetic players in oncology is therefore warranted, also with regard to the potential role of eRNAs and piRNAs in meningiomas (Figure 4).

The development of potential molecular therapies for patients with meningiomas is an important goal, particularly in light of the epigenetic aspects of this most common type of brain tumor in humans, as described in this review. Many molecular approaches have been pursued to date and are well documented in the literature; of particular note are investigational approaches using inhibitors of the mammalian target of rapamycin (mTOR), focal adhesion kinase (FAK), cyclin-dependent kinases (CDK), phosphoinositide-3-kinase (PI3K), and the Sonic Hedgehog signaling pathway [4,5,6,196]. Epigenetic approaches to meningiomas have so far been dominated by the use of histone deacetylase inhibitors (Table 3); however, approaches using inhibitors of DNA methyl transferases, histone methyltransferases, and Jumonji histone demethylases have also been tested [7,9]. Taken together, these epigenetic approaches demonstrate a significant inhibitory effect on meningioma cells in vitro and in mouse models (Table 3). The results of these studies underscore the broad scope of additional research opportunities presented by epigenetic mechanisms relevant to oncology. This applies equally to the epigenetic changes in meningiomas that are already known and to those that have not yet been studied in this type of tumor (Figure 4). In conclusion, the broad spectrum of epigenetic alterations studied to date and further epigenetic mechanisms that are potentially involved in the molecular pathology of meningiomas provide a promising basis for further approaches towards a molecular understanding of this most common type of human brain tumor. The central goal is to develop clinically applicable molecular therapies based on individualized treatment strategies that take into account the distinct molecular and epigenetic characteristics of individual tumor cases.

**Table 3:** Epigenetic approaches for potential molecular therapies of meningiomas—in vitro, experimental, or Phase 1 clinical trials; applied compound, type of the compound, references, observations during the study. **Abbreviations in alphabetical order:** DMTR: DNA-methyl transferase; HDAC: histone deacetylase; HMTR: histone methyl transferase; JHDM: Jumonji histone demethylase.

**Vorinostat** (Pan-HDAC-inhibitor) [1] Selective inhibition of the viability of meningioma cells from tumors with pronounced activation of cell cycle and proliferation signaling pathways; likewise, reduced tumor growth and prolonged survival in an intracranial xenograft model following administration of vorinostat
**AR-42** (Pan-HDAC-inhibitor) [51] This Phase 1 study examined a total of two NF2-associated meningiomas, as well as other NF2-associated tumors such as urothelial and breast carcinomas. Stable disease in 53% of patients is noted as the best response, and the compound is reported to be well tolerated
**Trichostatin A** (Pan-HDAC-inhibitor) [52] Enhancement of the infectivity and antitumor activity of an oncolytic herpes simplex virus therapy in a human meningioma mouse model. Similar effect observed following administration of panobinostat in the same study
**Cay10603** (selective HDAC6-inhibitor) [53] External radiation therapy leads to increased expression of HDAC6 in meningioma cells; in contrast, the combination of Cay10603 and radiation therapy results in synergistic inhibition of tumor growth through inhibition of the WNT/β-catenin signaling pathway and the c-MYC oncogene
**Panobinostat** (Pan-HDAC-inhibitor) [8] The compound with the highest anti-meningioma efficacy in a standardized patient-derived tumor organoid model; additional evidence of HDAC8 upregulation in organoids resistant to panobinostat
**Panobinostat** (Pan-HDAC-inhibitor) [7] Cell viability was reduced in 36 of 43 meningiomas; unlike the other epigenetic inhibitors tested (JIB-04, LAQ824, UNC0631), panabinostat was effective in most cases of meningiomas, particularly in those with tumor grade 1
**JIB-04** (JHDM-inhibitor) [7] In addition to panabinostat, JIB-04 was the second most common epigenetic inhibitor to cause a decline in cell viability in meningiomas; this demonstrates the importance of Jumonji histone demethylases in meningiomas as well
**LAQ824** (Pan-HDAC-inhibitor) [7] Unlike the other compounds studied, it was the most effective epigenetic inhibitor in grade 2 meningiomas
**UNC0631** (selective inhibitor of HMTR G9a) [7] The most effective compound in terms of reducing cell viability in the only case of grade 3 meningioma in the same study
**Decitabine** (DMTR-inhibitor) [9] A significant reduction in cell viability was observed in meningioma cell cultures across all tumor grades, in both primary and recurrent tumors

## Data Availability

No new data were created or analysed in this study.

## Abbreviations

**5hmC**	hydroxymethylation of cytosine at position 5
**5mC**	methylation of cytosine at position 5
**6mA**	methylation of adenine at position 6
**ACTB**	Actin beta
**ACTL6A/B**	Actin like 6A/B
**AKAP12**	A-kinase anchor protein 12
**AKT1**	AKT serine/threonine kinase 1
**ALKBH5**	alkylated DNA repair protein AlkB homolog 5
**AMH**	anti-mullerian hormone
**ANCR**	anti-differentiation ncRNA
**APOBEC3A**	apolipoprotein B mRNA editing catalytic polypeptide-like 3A
**ARID1A**	AT-rich interaction domain 1A
**ARID2**	AT-rich interaction domain 2
**BAF**	Brahma-associated factor complex
**BAK**	BCL-2 homologous antagonist/killer
**BCL2**	B-cell lymphoma 2 apoptosis regulator
**BCL7A**	BAF chromatin remodeling complex subunit 7A
**BRD7**	Bromodomain containing 7
**BRG1**	brahma-related gene 1
**BRM**	Brahma gene
**cBAF**	canonical Brahma-associated factor complex
**CCL21**	C-C motif chemokine ligand 21
**CCM**	clear cell meningioma
**CCND1**	Cyclin-D1
**CD8**	cluster of differentiation 8
**CDE3**	CD3 epsilon subunit of the T-cell receptor complex
**CDK4/6**	cyclin-dependent kinases 4/6
**CDKN2A**	cyclin-dependent kinase inhibitor 2A
**ciRNA**	circular RNA
**CKS2**	CDC28 protein kinase regulatory subunit 2
**COL5A1**	collagen alpha-1(V) chain
**CPEB3**	cytoplasmic polyadenylation element binding protein 3
**CpG sites**	cytosine–phosphate–guanine sites
**CTCF**	zinc-dependent CCCTC-binding factor
**DFP1**	Double PHD fingers 1
**DGCR8**	Di-George Syndrome Critical Region Gene 8
**DNA**	deoxyribonucleic acid
**EGFR**	epidermal growth factor receptor
**EHMT2**	euchromatic histone methyl transferase 2
**EIF4A3**	eukaryotic translation initiation factor 4A3
**ERK2**	extracellular signal-regulated kinase 2
**eRNA**	enhancer RNA
**EZH2**	enhancer of zeste homolog 2
**FASN**	fatty acid synthase
**FOXC1**	forkhead box C1
**FOXCUT**	forkhead box C1 upstream transcript
**FOXM1**	forkhead box family 1
**FRAT1**	frequently rearranged in advanced T-cell lymphomas
**FSCN1**	fascin actin-bundling protein 1
**GOLGA6A-1**	golgin A6 family member A
**GSTP1**	glutathione S-transferase Pi 1
**H3K27me3**	trimethylation of lysine at position 27 of histone 3
**H3K4me3**	trimethylation of lysine at position 4 of histone 3
**H3K9me2/3**	di- or trimethylation of lysine at position 9 of histone 3
**HAT**	histone acetyltransferase
**HCC**	hepatocellular carcinoma
**HDAC**	histone deacetylase
**HLA-DMA**	major histocompatibility complex, class II, DM alpha
**HLA-DPB1**	major histocompatibility complex, class II, DP Beta 1
**HMGB1**	high mobility group box 1
**HOX genes**	homeobox genes
**HOXA5**	homeobox cluster A gene number 5
**HuR**	human antigen R
**IDH**	isocitrate dehydrogenase
**IGF1**	insulin-like growth factor 1
**IMAT1**	Invasive meningioma-associated transcript 1
**INO80**	inositol-requiring mutant 80 complex
**ISLR2**	immunoglobulin superfamily containing leucine rich repeat 2
**ISWI**	imitation switch complex
**JAM3**	junctional adhesion molecule 3
**KDM6A/B**	lysine-specific demethylase 6A/B
**KLF4**	KLF transcription factor 4
**lncRNA**	long non-coding RNA
**LYVE1**	lymphatic vessel endothelial hyaluronan receptor 1
**LZTR1**	Leucine zipper-like post-translational regulator 1
**MAL2**	mal-T cell differentiation protein 2
**MALAT1**	Metastasis-associated lung adenocarcinoma transcript 1
**MC ben-1/2/3**	methylation class benign 1/2/3
**MC int-A/B**	methylation class intermediate A/B
**MC mal**	methylation class malignant
**MDM2**	MDM2 proto-oncogene
**MEG3**	maternally expressed 3
**miRNA**	micro-RNA
**MMP9**	matrix metalloproteinase 9
**mRNA**	messenger RNA
**mTOR**	mammalian target of rapamycin
**MYC**	MYC proto-oncogene
**NAD**	nicotinamide adenine dinucleotide
**ncBAF**	non-canonical Brahma-associated factor complex
**ncRNA**	non-coding RNA
**NF2**	neurofibromatosis type 2
**NF-κB**	nuclear factor kappa-light-chain-enhancer of activated B cells
**oHSV**	oncolytic herpes simplex virus
**P53**	tumor protein p53
**PADs**	peptidyl-arginine deiminases
**PBAF**	polybromo-Brahma-associated factor complex
**PBRM1**	polybromo-1
**PD-L1**	programmed death-ligand 1
**PENK**	proenkephalin
**PFS**	progression-free survival
**PHF10**	PHD finger protein 10
**PHH3**	phosphorylation of histone H3
**PI3K/AKT**	phosphatidylinositol 3-kinase and protein kinase B pathway
**PIK3CA**	phosphatidylinositol bisphosphate 3-kinase catalytic subunit alpha
**piRNA**	P-element-induced wimpy testis (PIWI) interacting RNA
**PRC2**	polycomb repressive complex 2
**PTEN**	phosphatase and tensin homolog
**PTX3**	pentraxin 3
**RAB14**	ras-related protein Rab-14
**RB1**	retinoblastoma transcriptional corepressor 1
**RISC**	RNA-induced silencing complex
**RNA**	ribonucleic acid
**RTPS1**	rhabdoid tumor predisposition syndrome type 1
**SHH**	sonic hedgehog signaling pathway
**SIRT1**	Sirtuin 1
**SMAD4**	SMAD Family Member 4
**SMARCA2/4**	SWI/SNF-related matrix-associated actin-dependent regulator of chromatin A2/4
**SMARCB1**	SWI/SNF-related matrix-associated actin-dependent regulator of chromatin B1
**SMO**	smoothened frizzled class receptor
**SNAIL1**	Snail Family Transcriptional Repressor 1
**SNF1/2**	sucrose non-fermentable 1/2
**SNHG1**	small nucleolar RNA host gene 1
**snoRNAs**	small nucleolar RNAs
**SS18**	SS18 subunit of BAF chromatin remodeling complex
**STAT3**	signal transducer and activator of transcription 3
**SUMO**	small ubiquitin-like modifier
**Survivin**	baculoviral IAP repeat containing 5
**SWI**	switched
**TCF12**	transcription factor 12
**TERRA**	telomeric repeat-containing RNA
**TERT**	telomerase reverse transcriptase
**TIMP3**	tissue inhibitor of metalloproteinase 3
**TP53**	tumor protein 53
**TP73**	tumor protein 73
**TRAF7**	TNF receptor-associated factor 7
**WNK2**	WNK lysine deficient protein kinase 2
**WNT**	wingless-int-1/β-catenin pathway
**ZEB1**	Zinc Finger E-Box Binding Homeobox 1
